# A Force Balanced Fragmentation Method for *ab Initio* Molecular Dynamic Simulation of Protein

**DOI:** 10.3389/fchem.2018.00189

**Published:** 2018-05-30

**Authors:** Mingyuan Xu, Tong Zhu, John Z. H. Zhang

**Affiliations:** ^1^State Key Lab of Precision Spectroscopy, Shanghai Engineering Research Center of Molecular Therapeutics & New Drug Development, Shanghai Key Laboratory of Green Chemistry & Chemical Process, School of Chemistry and Molecular Engineering, East China Normal University, Shanghai, China; ^2^NYU-ECNU Center for Computational Chemistry at New York University Shanghai, Shanghai, China; ^3^Department of Chemistry, New York University, New York, NY, United States; ^4^Collaborative Innovation Center of Extreme Optics, Shanxi University, Taiyuan, China

**Keywords:** quantum fragment method, *ab initio* molecular dynamics, force balanced, GB3, protein dynamics, MFCC

## Abstract

A force balanced generalized molecular fractionation with conjugate caps (FB-GMFCC) method is proposed for *ab initio* molecular dynamic simulation of proteins. In this approach, the energy of the protein is computed by a linear combination of the QM energies of individual residues and molecular fragments that account for the two-body interaction of hydrogen bond between backbone peptides. The atomic forces on the caped H atoms were corrected to conserve the total force of the protein. Using this approach, *ab initio* molecular dynamic simulation of an Ace-(ALA)_9_-NME linear peptide showed the conservation of the total energy of the system throughout the simulation. Further a more robust 110 ps *ab initio* molecular dynamic simulation was performed for a protein with 56 residues and 862 atoms in explicit water. Compared with the classical force field, the *ab initio* molecular dynamic simulations gave better description of the geometry of peptide bonds. Although further development is still needed, the current approach is highly efficient, trivially parallel, and can be applied to *ab initio* molecular dynamic simulation study of large proteins.

## Introduction

Molecular dynamic (MD) simulation plays an increasingly important role in the study of structural and dynamical properties of biomolecules at the atomic level (Karplus and Petsko, 1990; Cheatham and Kollman, 2000; Karplus and McCammon, 2002). With the ever-increasing power of computer hardware and the development of enhanced sampling methods, a significant advance in MD simulations with larger systems and longer simulation time have been achieved over the past decades (Shaw et al., 2010; Prinz et al., 2011). However, the accuracy and reliability of MD results are highly dependent on the accuracy of the force field employed in the simulation (Weiner et al., 1984, 1986; Ponder and Case, 2003). Despite widely successful applications of the current force fields in bio-molecular simulations, these simplified, pre-defined pairwise force fields have serious drawbacks. The most widely known deficiency is that the atomic charges in most of these force fields are pre-fixed, and there is no explicit treatment of electrostatic polarization and charge transfer (Duan et al., 2010; Tong et al., 2010; Ji and Mei, 2014). In the past few decades, significant efforts have been devoted to the development of polarizable force fields. However, although great achievements have been made, the accuracy of these polarizable force fields still have a lot of room for improvement. In addition, many force fields have a bias toward the secondary structure of the protein. For example, the α-helical propensity of the AMBER03 force field is too high relative to experimental measurements, while that of AMBER99SB is arguably too low (Best et al., 2008).

Compared with classical force fields, QM calculation can provide much more accurate potential energy function for the studied system, and include all important quantum effects. The advantage or need of the so-called *ab initio* molecular dynamic (AIMD) simulation over classical force fields in the study of proteins have been reported by various researchers (Wei et al., 2001; Dal Peraro et al., 2005; Ufimtsev and Martinez, 2009; Ufimtsev et al., 2011; Isborn et al., 2013). In these AIMD calculations, the atomic forces of the studied protein were calculated by QM methods, normally on the DFT level, whereas the motion of the nuclei was handled by classical mechanics. However, QM calculation needs a large amount of computational cost, which means that it can only be used for proteins with relatively small size.

So far, considerable efforts have been made to extend the applicability of QM calculation to large systems. Among existing approaches, the fragment-based QM methods has attracted much attention (Gordon et al., 2012; Collins et al., 2014; Li et al., 2014; Pruitt et al., 2014; Ramabhadran and Raghavachari, 2014; Chung et al., 2015; Collins and Bettens, 2015; Raghavachari and Saha, 2015). These approaches based on the chemical locality of molecular system, which assumes that the local regions of a molecular system can only be influenced weakly by atoms that are far away from it (Xu et al., 1998; Fedorov et al., 2014; Gao et al., 2014; He et al., 2014; Pruitt et al., 2014). In this kind of methods, the studied system is divided into small subsystems (fragments); the properties of these fragments such as energy are calculated separately by QM method. Then the property of the whole system can be obtained by taking a proper combination of the properties of these individual fragments. The fragment-based QM method is attractive in several aspects, such as easy implementation of parallelization without extensively modifying the existing QM programs and can be combined with all levels of *ab initio* electronic structure theories. In our previous study (Liu et al., 2015), a fragment based approach is presented for AIMD simulation of protein. In this approach, the potential energy and atomic forces of the studied protein are calculated by a recently developed electrostatically embedded generalized molecular fractionation with conjugate caps (EE-GMFCC) method (Wang et al., 2013). This AIMD approach had been applied to MD simulation of a small benchmark protein Trpcage in both gas phase and in solution. Compared with AMBER force field, this method can give more stable protein structure in simulation, and capture quantum effects that are missing in standard classical MD simulations.

To further improve the accuracy and efficiency of the AIMD simulations for protein, in this work, we presented a force balanced generalized molecular fractionation with conjugate caps (FB-GMFCC) method and checked its performance in the AIMD simulations for several systems. The paper is organized as follows. The next section provides a description of the FB-GMFCC approach. In section Result and Discussion, we performed AIMD simulations on two selected proteins to validate the new method, and finally, a brief summary will be given in section Conclusion.

## Theory and method

The FB-GMFCC method was developed based on the framework of molecular fractionation with conjugate caps (MFCC) approach (Zhang and Zhang, 2003). The computation procedure of FB-GMFCC can be roughly divided into two steps. Firstly, the given protein is cut into caped molecular fragments, including individual residues and residues that form backbone hydrogen bonds. Then the energy and atomic forces of each fragment are calculated by QM methods separately. Secondly, the AMOEBA polarizable force field (Ren and Ponder, 2002; Ponder et al., 2010; Ren et al., 2011; Wu et al., 2012) was employed to describe the long-range non-bonded interactions. Thus, the total energy of the protein system is obtained by a summation of quantum and classical components,

(1) $$\begin{array}{l} {\text{E}}_{\text{FB }-\text{GMFCC}}={\text{E}}_{\text{QM}}+{\text{E}}_{\text{MM}} \end{array}$$

Computational details of these energy components are describe below.

### Calculation of E_QM_

**Figure 1:**
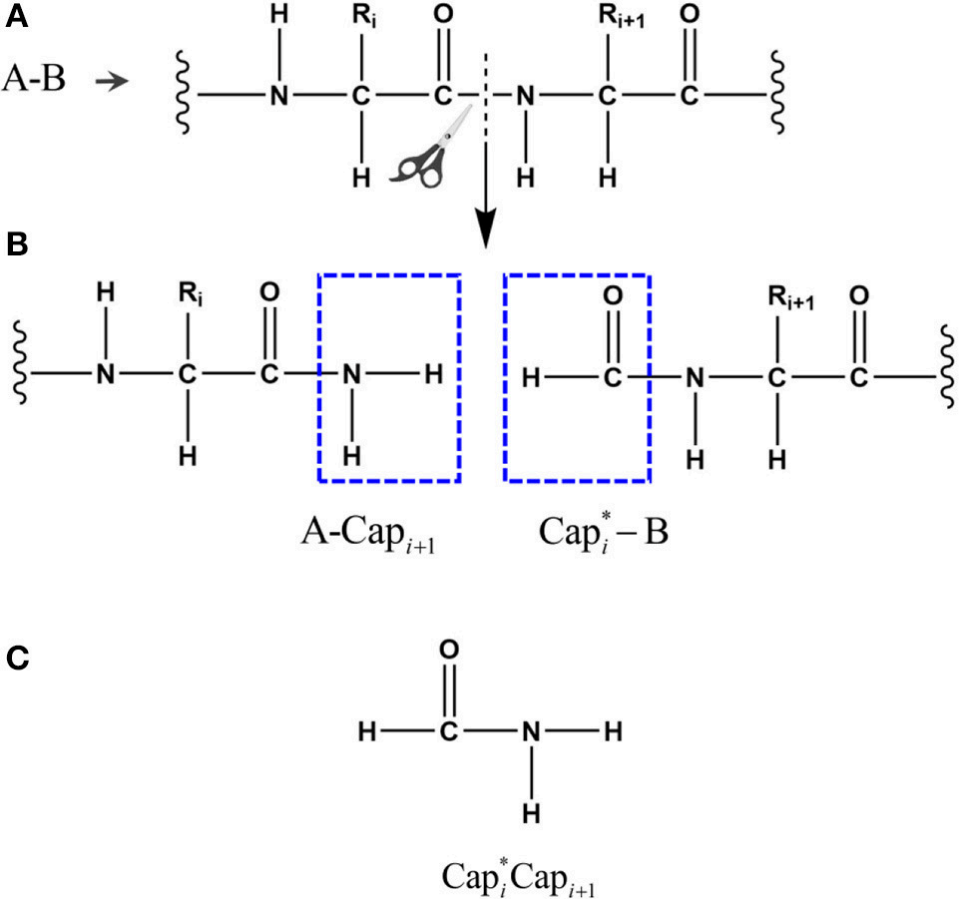
The peptide bond is cut in the upper panel **(A)** and the fragments are capped with ${\text{Cap}}_{i+1}^{}$ and it's conjugate ${\text{Cap}}_{i}^{*}$ in the middle panel **(B)**, where *i* represents the index of *i*th amino acid in the given protein. The atomic structure of the conjugate caps is shown in the bottom panel **(C)**, defined as the fused molecular species.

To calculate the energy E_QM_, a given protein with N amino acids (defined as A_1_A_2_A_3_ … A_N_) is decomposed into N individual fragments by cutting through the peptide bonds (Figure 1). At every cut point, a pair of molecular caps were designed to saturate each fragment in order to preserve the local chemical environment. To minimize the computational cost, we simply use the amine and formyl group from the peptide bond as molecular caps, which are conjugate to each other (by forming a peptide bond) and are much smaller than that used in the EE-GMFCC approach. To avoid dangling bond, hydrogen atoms were added to terminate the molecular cap, the position of these extra H atoms are determined from the coordinates of the corresponding C_α_ atoms.

**Figure 2:**
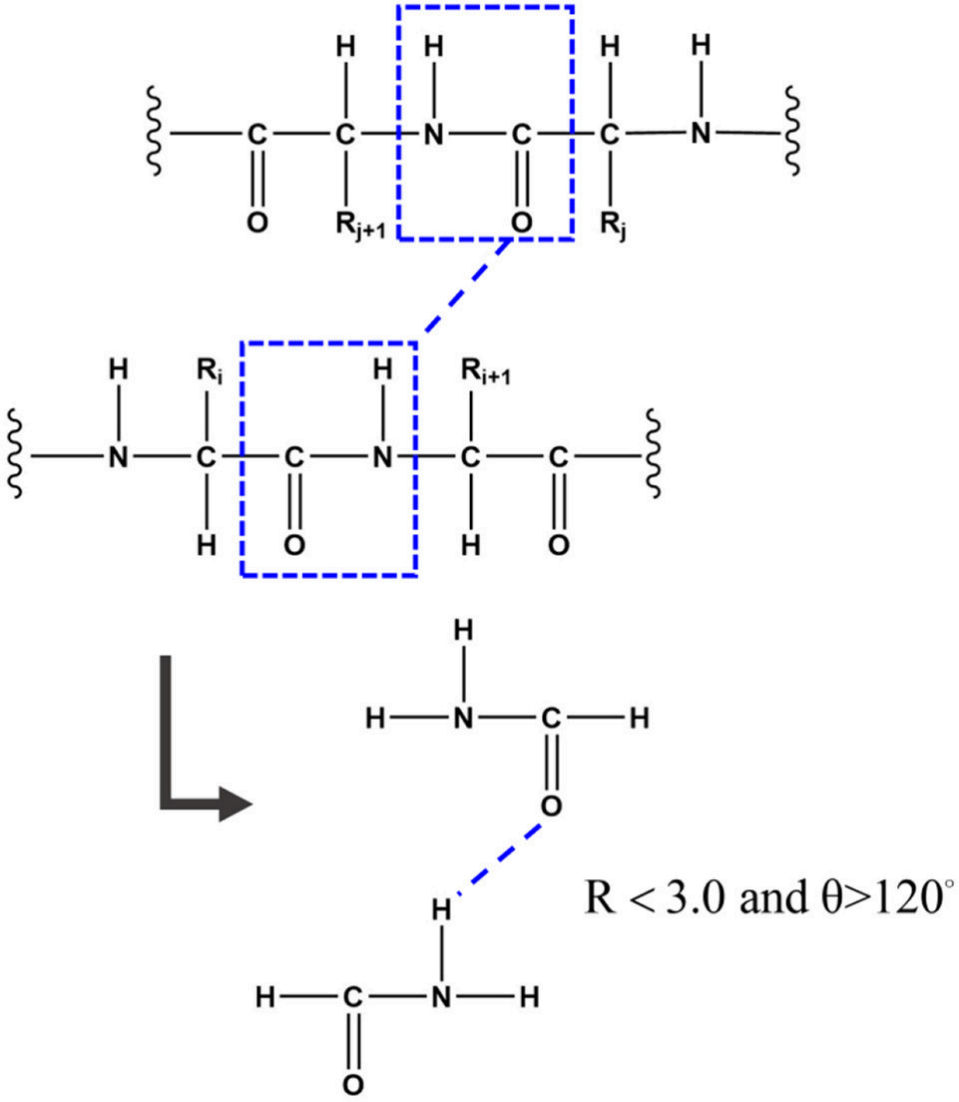
To better describe the backbone hydrogen bond, if the distance between the donor H atom and the acceptor O atom is <3.0 Å and the angle of N-H^N^-O is larger than 120°, the 2-Body correction will be considered. To reduce computational cost, only the H-saturated peptide bond which contains the donor or the accepter was kept in the two-body QM calculation.

Hydrogen bond is one of the most important structural elements of protein and the dominant factor that stabilizes the protein secondary structures. Many previous works demonstrated that the strength of hydrogen bond from simulations under non-polarizable force fields is underestimated due to the lack of polarization effect (Ji et al., 2008; Gao et al., 2012). In the FB-GMFCC method, the backbone hydrogen bond was considered by two-body QM calculation. To reduce computational cost, only the H-saturated peptide bond which contains the donor or the accepter (which actually is a formamide as shown in Figure 2) was kept in the two-body QM calculation, the position of the extra H atoms are also determined from the coordinates of the corresponding C_α_ atoms. If the distance between donor H atom and acceptor O atom is <3.0 Å and the angle θ of N-H_N_-O is larger than 120°, the 2-body correction will be considered. Thus, E_QM_ can be expressed by the following formula:

(2) $$\begin{array}{l} {\text{E}}_{\text{QM}}={\text{E}}_{\text{fragment}}-{\text{E}}_{\text{concap}}+{\text{E}}_{\text{two-body}}\\ \;=\sum_{i=2}^{N-1}\text{E}({\text{Cap}}_{i-1}^{*}{\text{A}}_{i}{\text{Cap}}_{i+1})-\sum_{i=2}^{N-2}\text{E}({\text{Cap}}_{i}^{*}{\text{Cap}}_{i+1})\\ \;+\sum_{\begin{array}{c} i,j>i+2\\ |{\text{R}}_{{\text{H}}_{\text{N}-\text{O}}}|\leq \text{λ}\\ {∠}_{\text{N}-{\text{H}}_{\text{N}}-\text{O}}\geq \theta \end{array}}\left(\text{E}({\text{A}}_{i}^{p}{\text{A}}_{j}^{p})-\text{E}({\text{A}}_{i}^{p})-\text{E}({\text{A}}_{j}^{p})\right) \end{array}$$

Where the *i* and *j* represent the index of *i*th and *j*th residues, respectively. If the formyl or amide group of residue A is included in a backbone hydrogen bond, A^p^ represents the H-saturated peptide bond which contains this group. The first term E(Cap${}_{\text{i}-1}^{*}$A_*i*_Cap_*i*+1_) in Equation (2) represents the self-energy of fragment *i* (the *i*th residueA_*i*_capped with a left cap ${\mathrm{Cap}}_{i-1}^{*}$ and a right cap Cap_*i*+1_). And it is clear that the self-energy of conjugate caps E(Cap^*^_i_Cap_*i*+1_) are double counted in first term of Equation (2) and it should be deducted.

### Calculation of E_MM_

The E_QM_ term includes the self-energy of individual residue and the two-body correction of the interaction energy between residues that form backbone hydrogen bonds. To obtain the total energy expression for proteins, the classical force field was introduced to represent the long-range non-bonded interactions. In our previous study, we found that the electrostatic polarization arising from the environment also plays a critical role for including the many body effect in fragmentation methods (Wang et al., 2013). To describe the electrostatic polarization effect efficiently, we employed the polarizable atomic multipole-based AMOEBA force field (Ren and Ponder, 2002; Ponder et al., 2010; Ren et al., 2011; Wu et al., 2012). The expression of E_MM_ is as the following:

(3) $${\text{E}}_{\text{MM}}=\sum_{{}_{\begin{array}{c} i,j\notin same\\ \text{QM zone} \end{array}}}{\text{E}}_{\text{ele}}^{\text{perm}}(i,j)+{\text{E}}_{\text{ele}}^{\text{ind}}(i,j)+{\text{E}}_{\text{vdW}}(i,j)$$

Details about calculations of Van der Waals interactions, permanent and induced electrostatic energies of the AMOEBA force field could be found in Refs (Ren and Ponder, 2002; Ponder et al., 2010; Ren et al., 2011; Wu et al., 2012). For any two atoms that have not been calculated in the same QM zone, these non-neighboring interactions between them should be added to the total energy expression.

### Balance the force

To obtain atomic forces, we need to compute the derivative of FB-MFCC with respect to nuclear coordinates. For a given atom *m*, the atomic force can be expressed as following:

(4) $$\begin{array}{r} {\text{F}}_{m}=-\nabla_{m}{\text{E}}_{\text{FB – GMFCC}}=-\nabla_{m}{\text{E}}_{\text{QM}}-\nabla_{m}{\text{E}}_{\text{MM}} \end{array}$$

**Figure 3:**
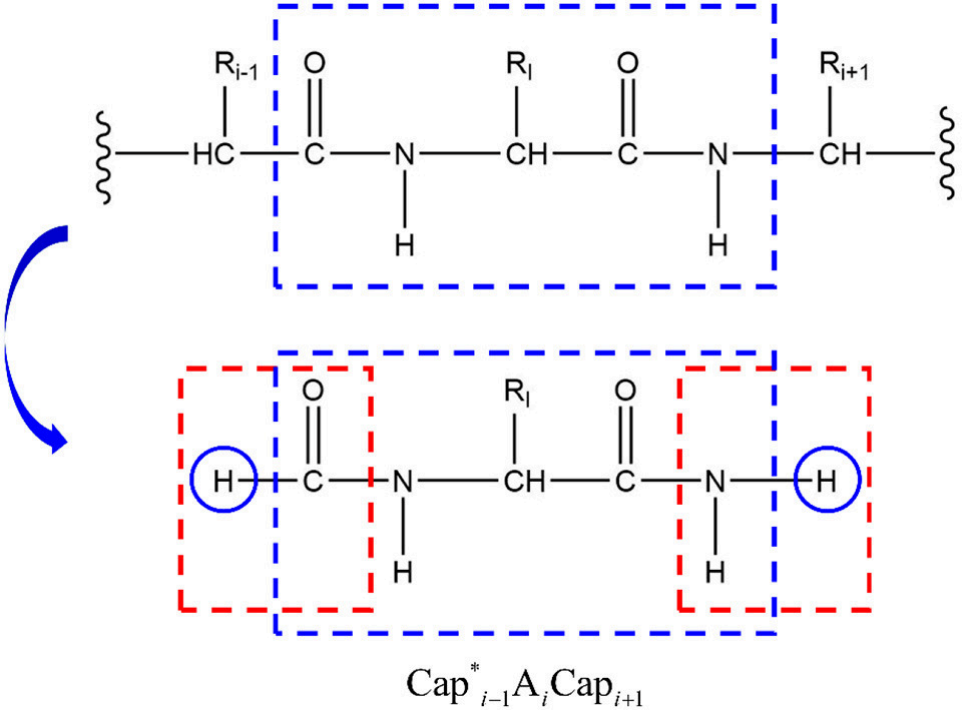
Extra hydrogen atoms (shown in blue cycles) are added to avoid dangling bonds.

It should be noted, however, that we employed extra hydrogen atoms to avoid dangling bonds (Figure 3) and their coordinates were determined from those of the corresponding C_α_ atoms. Because the forces on these extra hydrogen atoms in capped fragments E(Cap${}_{\text{i}-1}^{*}$A_*i*_Cap_*i*+1_) cannot be canceled exactly by subtracting those in the caps E(Cap^*^_i_Cap_*i*+1_), it will not exactly conserve the energy. In order to fix this problem, we balance the forces on the corresponding C_α_ atoms by adding the differences of forces on these extra hydrogen atoms. For instance, the difference of the forces of the H atoms added to the carbonyl group of the residue i−1 (H atom in the left blue cycle of Figure 3) is

(5) $$\begin{array}{r} \Delta \text{F}={\text{F}}_{{\text{Cap}}_{i}}^{\text{ex-H}}({\text{Cap}}_{i-1}^{*}{\text{A}}_{i}{\text{Cap}}_{i+1})-{\text{F}}_{{\text{Cap}}_{i}}^{\text{ex-H}}({\text{Cap}}_{i-1}^{*}{\text{Cap}}_{i})\neq 0, \end{array}$$

Which is added to the force of the C_α_ atom of residue *i*−1,

(6) $$\begin{array}{r} {\text{F}}_{C_{\alpha }}^{\text{final}}={\text{F}}_{C_{\alpha }}+\Delta \text{F} \end{array}$$

This approach will balance the forces of the fragments and conserve the total energy of the system.

## Result and discussion

### Performance of FB-GMFCC on pure proteins

To validate the FB-GMFCC method, we checked its performance for four protein systems and compared the calculated results with that calculated by conventional full-system QM calculations. An Ace-(ALA)_9_-NME linear peptide was constructed by the TLEAP software in the AMBER16 package, and three small proteins with different secondary structures are selected from the protein data bank (ID: 2I9M, 1LE1, and 2OED). Energy minimization (by using gradient descent and conjugate gradient algorithms with Amber ff14SB force field) was performed to remove bad contacts in these structures before QM calculations. Comparisons of job CPU times of FB-GMFCC and full system QM calculations were shown in Table 1. We can see that FB-GMFCC method is 4 or 5 times faster than full QM calculation for a real protein with about 200 atoms. For the larger 2OED protein, the full-system QM calculation is not possible on our computer system due to its large size. It should be noted that the computational time in the present approach is essentially linear with the system size as shown in Table 1.

**Table 1:** Comparison of the computational cost of FB-GMFCC and full-system QM calculation on the Linux server with two Intel E5-2680v3 CPUs (14 cores, 2.50 GHz).

**System**	**Protein type**	**Atom No**.	**CPU time (hour)**	
			**Full-system QM**	**FB-GMFCC**
Ace-(ALA)_9_-NME	Linear peptide	102	0.63	0.27
2I9M	α-helix	237	4.97	0.90
1LE1	β-sheet	194	4.06	0.76
2OED	Mixed structure	862	N/A*	3.16

All calculations were performed with GAUSSIAN09 at M062X/6-31G^*^ level. ^*^Full-system QM calculation is not possible on our current machine due to large size of the system.

**Figure 4:**
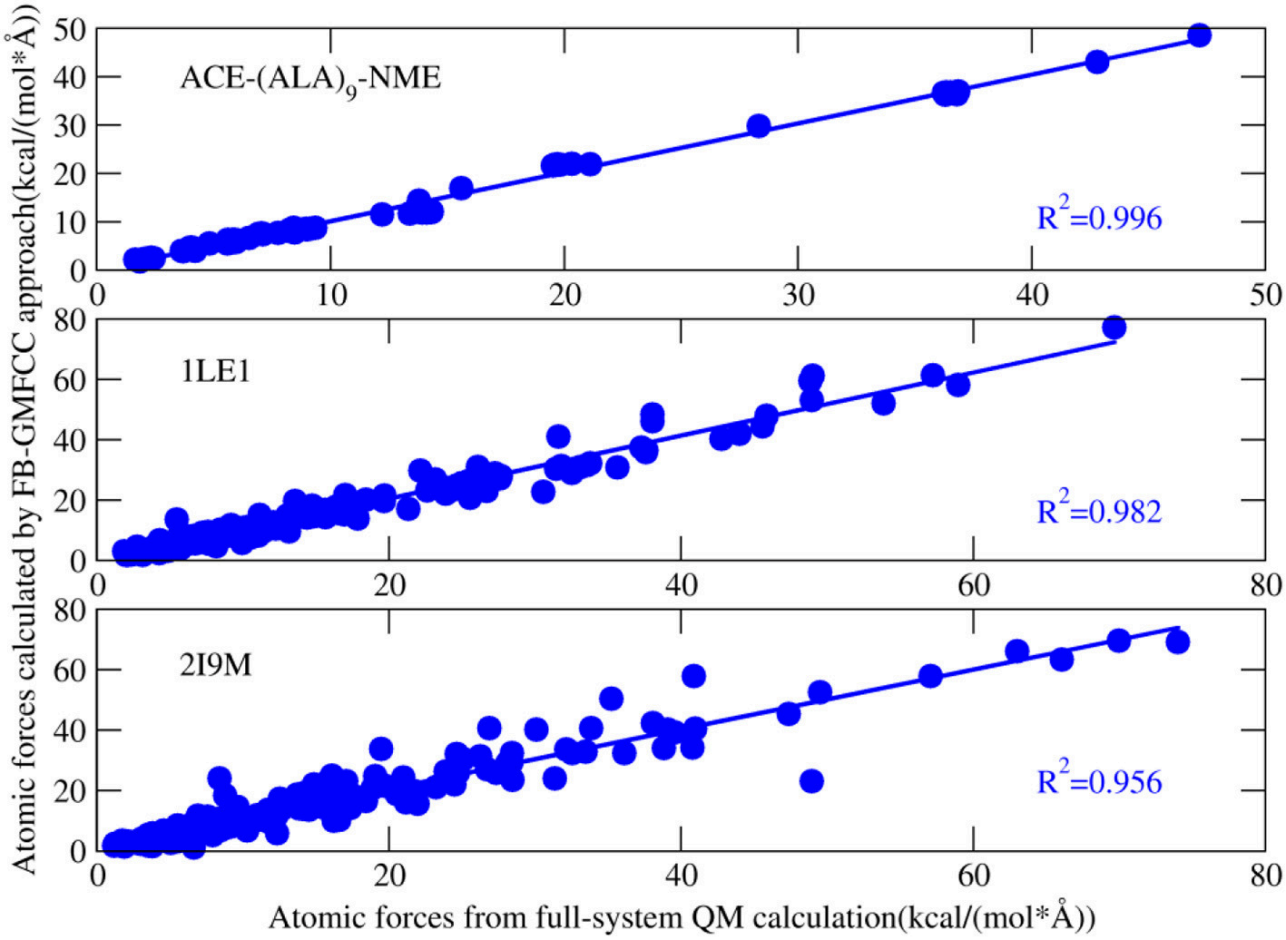
Correlation between atomic forces of three proteins [ACE-(ALA)_9_-NME linear peptide, 1LE1, 2I9M] calculated by full-system QM calculation and that calculated by FB-MFCC. All the QM calculations were performed at the M062X/6-31G* level.

Figure 4 shows the comparison of computed atomic forces with those from full-system QM calculation. Overall, the atomic forces are in good agreement with the full-system calculations except a few points. For example, there is a bad point in the calculated atomic force of 2I9M (Figure 4), which correspond to one H atom on the ε-amino group of LYS8. After carefully checking the structure, we found that a salt bridge is formed between this group and the side chain of GLU4. As a result, this salt bridge cannot be accurately described by the force field which is used in the present method to describe interaction between non-neighboring residues.

### *Ab initio* molecular dynamic in gas phase

**Figure 5:**
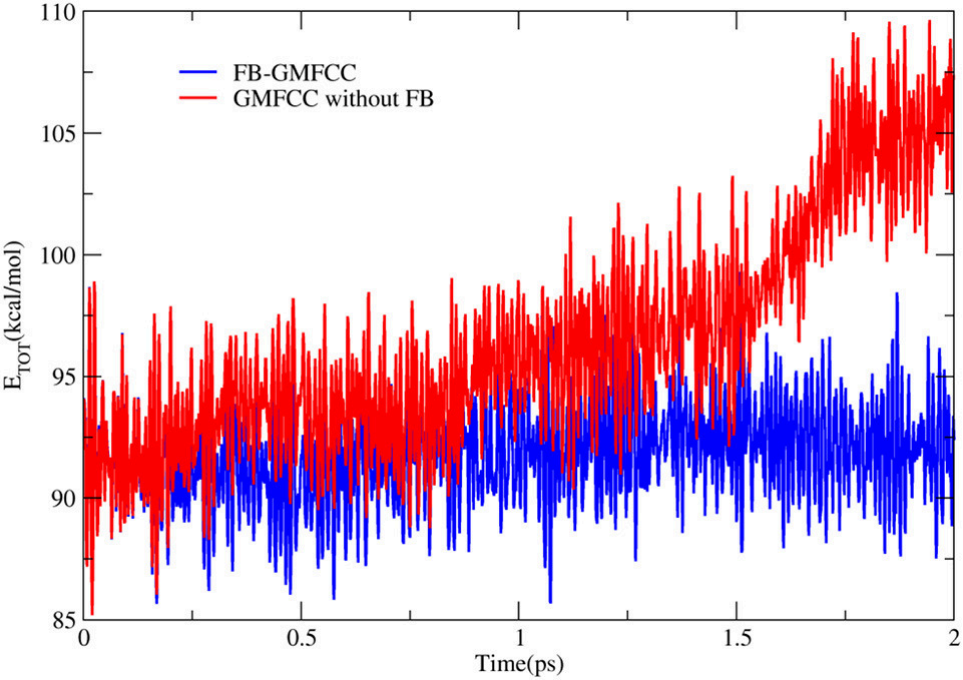
Time evolution of total energies in AIMD simulations of a linear peptide ACE-(ALA)_9_-NME with FB-GMFCC method (blue) and GMFCC without force balance (red) in gas phase and NVE ensemble.

To further check the performance of FB-GMFCC method, we performed an AIMD simulation for the linear peptide ACE-(ALA)_9_-NME in the NVE ensemble and gas phase. The simulation was performed by combining the FB-GMFCC and the TINKER program. Before the AIMD simulation, an energy minimization (by using gradient descent and conjugate gradient algorithms with Amber ff14 force field), a 400 ps heating simulation which heated the system from 0 to 300 K and a 5 ns equilibrium MD simulation (by using velocity verlet algorithm and the same force field) were performed. The AIMD simulation lasted 2 ps with 1fs time step and without any constraints. Another AIMD simulation without force balance was also performed as a reference. The total energy fluctuations in these two AIMD simulations were shown in Figure 5. As can be seen, the total energy in the AIMD simulation based on GMFCC without force balance is gradually increased in gas phase NVE ensemble, which means that the energy is not conserved if atomic forces of extra cap hydrogen atoms are not compensated to corresponding C_α_ atoms. However, the total energy of FB-GMFCC can be maintained well and conserved at 91 kcal/mol. On average, the extra H atoms can import extra forces as large as 11 kcal/(mol^*^ Å) to the system, which lead to additional works on the system. Thus it is necessary to balance the atomic forces on these extra H atoms in the AIMD simulation.

### AIMD in explicit water

Since water plays an important role in protein structure and dynamics, the study of protein should be carried out in the solvent environment. The FB-GMFCC approach can also be used to perform AIMD simulations for proteins in explicit solvent environment. The total energy of protein-solvent system with FB-GMFCC can be expressed by the following formula.

(7) $$\begin{array}{r} \begin{array}{c} {\text{E}}_{\text{total}}={\text{E}}_{\text{Protein}}^{\text{FB-GMFCC}}+{\text{E}}_{\text{water}}^{\text{MM}}+{\text{E}}_{\text{Protein-water}}^{\text{MM}} \end{array} \end{array}$$

To save the computation cost, the inter- and intra-interactions of water molecules and their interactions with proteins are described by classical force field (Amber ff14SB), as mechanical embedding in the QM/MM framework.

We performed 110 ps AIMD simulation for the relatively larger protein (2OED, 56 residues, 862 atoms) in explicit water. The protein was solvated in a water ball consisting of 3084 TIP3P water molecules. Before AIMD simulation, energy minimizations were performed to remove bad contacts in the system, and a 25 ps heating simulation was performed to heat the system slowly to 300 K. A restraint of 50 kcal/mol was used on the backbone to avoid large unphysical structural change in heating process. Then the system underwent AIMD simulation with 1fs time step and without any constraints. The Langevin thermostat with the collision frequency 2.0 ps^−1^ was applied to control the temperature. In addition, there was a 20 kcal/mol half-harmonic restrain used on the boundary of water ball to avoid the escaping of water molecules.

**Figure 6:**
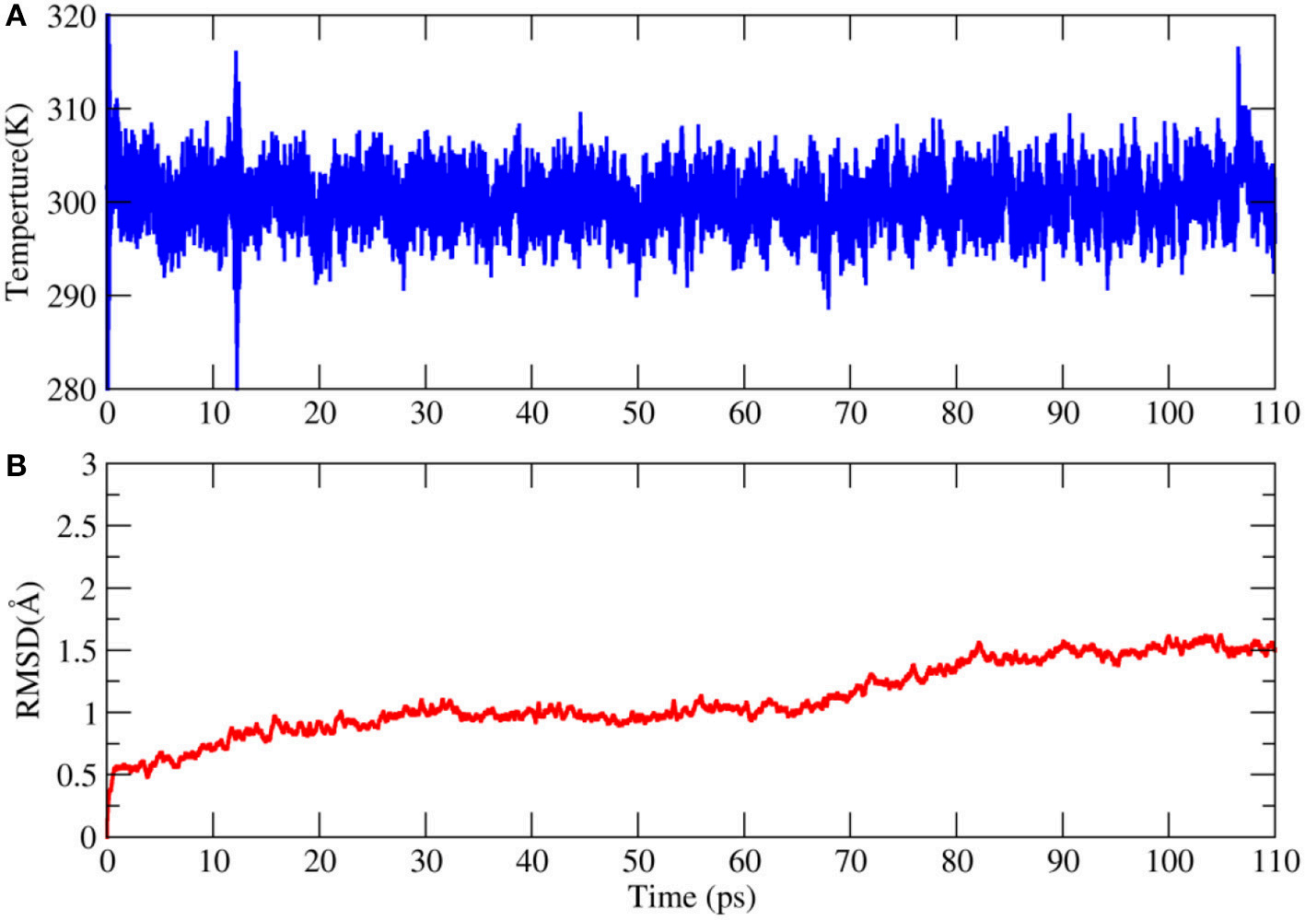
Time evolution of the temperature **(A)** and root mean square deviation (RMSD) of backbone atoms with respect to the X-ray structure in the AIMD simulation of 2OED protein **(B)**.

This AIMD simulation was performed on a linux server cluster with 30 nodes and each node has dual Intel Xeon E5-2680v3 CPUs. To balance the computational cost and accuracy, the combination of BLYP functional and 6-31G^*^ basis set was used in the calculation. It took 55 days to complete the simulation. The time evolution of temperature in the simulation was shown in Figure 6. We can see that the temperature is very stable in the trajectory. The backbone RMSD with respect to the X-ray structure is no larger than 1.5 Å, which means that the structure of 2OED protein are relatively stable during the110 ps AIMD simulation.

**Figure 7:**
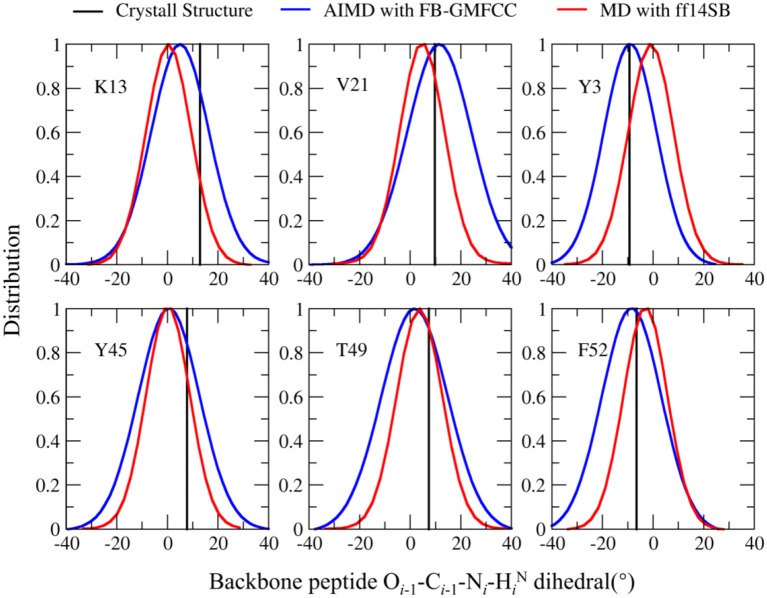
Comparison of the O_*i*−1_-C_*i*−1_-N_*i*_-H_*i*_^N^ dihedral angle distribution in the FB-GMFCC AIMD (blue lines) and classical MD simulations (with Amber ff14SB force field, red lines) for 6 selected peptide bonds. Black lines are experimental values.

Recently, many researchers discovered that considerable deviations from planarity of peptide bond (ω = 180°) can be identified in atomic resolution X-ray structures, sometimes even exceeding 10–15° (Wlodawer et al., 1984; MacArthur and Thornton, 1996; Ulmer et al., 2003). As the resolution of the structure (2OED) used in this work is very high (1.1 Å) and the coordinates of hydrogen atoms in this structure were further refined with NMR experiment (Ulmer et al., 2003), it is worth to compare the planarity of peptide bonds in the experimental structure and the AIMD trajectory. Six peptide bonds were selected from the experimental structure as their O_*i*−1_-C_*i*−1_-N_*i*−_H_*i*_^N^ dihedral angles deviate the most from the peptide plane. The result can be found in Table 2 and Figure 7. We can see that four of the six peptide bonds still maintained their large deviations. The results calculated by FB-GMFCC generally agree well with the experiments, especially for VAL21, TYR3, and PHE52. For comparison, we also test the performance of MD with classical force field (Amber ff14SB) at the same conditions. Not surprisingly, the Amber ff14SB force field prefers planer peptide bonds, which was predefined. As based on QM calculation, the AIMD simulation generally describes the intra-protein interactions more accurately.

**Table 2:** Comparison of six selected O_*i*−1_-C_*i*−1_-N_*i*−_H_*i*_^N^ dihedral angles in both the AIMD and AMBER MD calculations with experimental measurements.

**Residue**	**O**_**i−1**_**-C**_**i−1**_**-N**_**i−**_**H*****_*****i*****_***^**N**^ **dihedral (**^**°**^**)**		
	**Crystall structure**	**AIMD with FB-GMFCC**	**AMBER MD with ff14SB**
LYS13	12.88	5.91	1.84
VAL21	9.69	11.91	6.27
TYR3	−9.34	−8.23	0.74
TYR45	7.70	1.21	1.64
THR49	7.36	2.36	5.32
PHE52	−6.54	−7.72	−1.50

*The calculated values were averaged from the trajectories*.

## Conclusion

In this study, a force balanced generalized molecular fractionation with conjugate caps (FB-GMFCC) method was presented. In this approach, fragment-based energies of individual residues and interaction energies of residues that form backbone hydrogen bonds are calculated by quantum mechanics. Other non-bonded interactions are considered by the polarizable AMOEBA force field. The calculated atomic forces of this method showed good agreements with that calculated by the conventional full-system QM calculations. A key element of the FB-GMFCC method is that the atomic forces of capped H atoms are corrected to achieve the conservation of the total force of the studied system.

We also demonstrated the applicability of FB-GMFCC method for performing *ab initio* molecular dynamic (AIMD) simulations for proteins. The results of an Ace-(ALA)_9_-NME linear protein showed that only the balanced force can keep the conservation of the total energy during the simulation. An 110 ps AIMD simulation was also performed for a relatively large protein with 56 residues and 862 atoms in explicit water. Compared with the classical force field, the AIMD simulations gave better description about the geometry of peptide bonds. It should be note that the accuracy of the FB-MFCC method still have room to be improved. Further development of this method will focus on the consideration of strong short-range interactions such as salt bridges and hydrogen bond including side chains, relevant work is underway in our laboratory.

These results have shown that the FB-GMFCC approach is potentially powerful and attractive for studying protein dynamics. As a fragment based approach, the FB-GMFCC method is linear-scaling and trivially parallelizable. With further development and improvment, this method will become more and more practical for AIMD simulation of larger proteins.

## Author contributions

MX performed theoretical calculation and analysis of result. TZ developed the theory and contributed to the writing and discussion of the paper. JZ organized the project and contributed to the discussion and writing of the paper.

### Conflict of interest statement

The authors declare that the research was conducted in the absence of any commercial or financial relationships that could be construed as a potential conflict of interest.

## References

[B1] Best R. B. Buchete N.-V. Hummer G. (2008). Are current molecular dynamics force fields too helical? Biophys. J. 95, L07–L09. 10.1529/biophysj.108.132696 18456823 PMC2426634

[B2] Cheatham T. E. III. Kollman P. A. (2000). Molecular dynamics simulation of nucleic acids. Annu. Rev. Phys. Chem. 51, 435–471. 10.1146/annurev.physchem.51.1.435 11031289

[B3] Chung L. W. Sameera W. M. C. Ramozzi R. Page A. J. Hatanaka M. Petrova G. P. (2015). The ONIOM method and its applications. Chem. Rev. 115, 5678–5796. 10.1021/cr5004419 25853797

[B4] Collins M. A. Bettens R. P. A. (2015). Energy-based molecular fragmentation methods. Chem. Rev. 115, 5607–5642. 10.1021/cr500455b 25843427

[B5] Collins M. A. Cvitkovic M. W. Bettens R. P. A. (2014). The combined fragmentation and systematic molecular fragmentation methods. Acc. Chem. Res. 47, 2776–2785. 10.1021/ar500088d 24972052

[B6] Dal Peraro M. Raugei S. Carloni P. Klein M. L. (2005). Solute-solvent charge transfer in aqueous solution. ChemPhysChem 6, 1715–1718. 10.1002/cphc.200500039 16080223

[B7] Duan L. L. Mei Y. Zhang D. Zhang Q. G. Zhang J. Z. H. (2010). Folding of a helix at room temperature is critically aided by electrostatic polarization of intraprotein hydrogen bonds. J. Am. Chem. Soc. 132, 11159–11164. 10.1021/ja102735g 20698682

[B8] Fedorov D. G. Asada N. Nakanishi I. Kitaura K. (2014). The use of many-body expansions and geometry optimizations in fragment-based methods. Acc. Chem. Res. 47, 2846–2856. 10.1021/ar500224r 25144610

[B9] Gao J. Truhlar D. G. Wang Y. Mazack M. J. M. Loeffler P. Provorse M. R. (2014). Explicit polarization: a quantum mechanical framework for developing next generation force fields. Acc. Chem. Res. 47, 2837–2845. 10.1021/ar5002186 25098651 PMC4165456

[B10] Gao Y. Lu X. Duan L. L. Zhang J. Z. H. Mei Y. (2012). Polarization of intraprotein hydrogen bond is critical to thermal stability of short helix. J. Phys. Chem. B 116, 549–554. 10.1021/jp208953x 22126129

[B11] Gordon M. S. Fedorov D. G. Pruitt S. R. Slipchenko L. V. (2012). Fragmentation methods: a route to accurate calculations on large systems. Chem. Rev. 112, 632–672. 10.1021/cr200093j 21866983

[B12] He X. Zhu T. Wang X. Liu J. Zhang J. Z. H. (2014). Fragment quantum mechanical calculation of proteins and its applications. Acc. Chem. Res. 47, 2748–2757. 10.1021/ar500077t 24851673

[B13] Isborn C. M. Mar B. D. Curchod B. F. E. Tavernelli I. Martinez T. J. (2013). The charge transfer problem in density functional theory calculations of aqueously solvated molecules. J. Phys. Chem. B. 117, 12189–12201. 10.1021/jp4058274 23964865

[B14] Ji C. G. Mei Y. Zhang J. Z. H. (2008). Developing polarized protein-specific charges for protein dynamics: MD free energy calculation of pK(a) shifts for Asp(26)/Asp(20) in thioredoxin. Biophys. J. 95, 1080–1088. 10.1529/biophysj.108.131110 18645195 PMC2479593

[B15] Ji C. Mei Y. (2014). Some practical approaches to treating electrostatic polarization of proteins. Acc. Chem. Res. 47, 2795–2803. 10.1021/ar500094n 24883956

[B16] Karplus M. McCammon J. A. (2002). Molecular dynamics simulations of biomolecules. Nat. Struct. Biol. 9, 646–652. 10.1038/nsb0902-646 12198485

[B17] Karplus M. Petsko G. A. (1990). Molecular dynamics simulations in biology. Nature 347, 631–639. 10.1038/347631a0 2215695

[B18] Li S. Li W. Ma J. (2014). Generalized energy-based fragmentation approach and its applications to macromolecules and molecular aggregates. Acc. Chem. Res. 47, 2712–2720. 10.1021/ar500038z 24873495

[B19] Liu J. F. Zhu T. Wang X. W. He X. Zhang J. Z. H. (2015). Quantum fragment based *ab initio* molecular dynamics for proteins. J. Chem. Theor. Comput. 11, 5897–5905. 10.1021/acs.jctc.5b00558 26642993

[B20] MacArthur M. W. Thornton J. M. (1996). Deviations from planarity of the peptide bond in peptides and proteins. J. Mol. Biol. 264, 1180–1195. 10.1006/jmbi.1996.0705 9000639

[B21] Ponder J. W. Case D. A. (2003). Force fields for protein simulations. Protein Simulat. 66, 27–85. 10.1016/S0065-3233(03)66002-X 14631816

[B22] Ponder J. W. Wu C. Ren P. Pande V. S. Chodera J. D. Schnieders M. J. (2010). Current status of the AMOEBA polarizable force field. J. Phys. Chem. B 114, 2549–2564. 10.1021/jp910674d 20136072 PMC2918242

[B23] Prinz J. H. Wu H. Sarich M. Keller B. Senne M. Held M. (2011). Markov models of molecular kinetics: generation and validation. J. Chem. Phys. 134:174105 10.1063/1.3565032 21548671

[B24] Pruitt S. R. Bertoni C. Brorsen K. R. Gordon M. S. (2014). Efficient and accurate fragmentation methods. Acc. Chem. Res. 47, 2786–2794. 10.1021/ar500097m 24810424

[B25] Raghavachari K. Saha A. (2015). Accurate composite and fragment-based quantum chemical models for large molecules. Chem. Rev. 115, 5643–5677. 10.1021/cr500606e 25849163

[B26] Ramabhadran R. O. Raghavachari K. (2014). The successful merger of theoretical thermochemistry with fragment-based methods in quantum chemistry. Acc. Chem. Res. 47, 3596–3604. 10.1021/ar500294s 25393551

[B27] Ren P. Ponder J. W. (2002). Consistent treatment of inter- and intramolecular polarization in molecular mechanics calculations. J. Comput. Chem. 23, 1497–1506. 10.1002/jcc.10127 12395419

[B28] Ren P. Wu C. Ponder J. W. (2011). Polarizable atomic multipole-based molecular mechanics for organic molecules. J. Chem. Theor. Comput. 7, 3143–3161. 10.1021/ct200304d PMC3196664 22022236

[B29] Shaw D. E. Maragakis P. Lindorff-Larsen K. Piana S. Dror R. O. Eastwood M. P. (2010). Atomic-level characterization of the structural dynamics of proteins. Science 330, 341–346. 10.1126/science.1187409 20947758

[B30] Tong Y. Mei Y. Li Y. L. Ji C. G. Zhang J. Z. H. (2010). Electrostatic polarization makes a substantial contribution to the free energy of avidin-biotin binding. J. Am. Chem. Soc. 132, 5137–5142. 10.1021/ja909575j 20302307

[B31] Ufimtsev I. S. Luehr N. Martinez T. J. (2011). Charge transfer and polarization in solvated proteins from *ab initio* molecular dynamics. J. Phys. Chem. Lett. 2, 1789–1793. 10.1021/jz200697c

[B32] Ufimtsev I. S. Martinez T. J. (2009). Quantum chemistry on graphical processing units. 3. analytical energy gradients, geometry optimization, and first principles molecular dynamics. J. Chem. Theor. Comput. 5, 2619–2628. 10.1021/ct9003004 26631777

[B33] Ulmer T. S. Ramirez B. E. Delaglio F. Bax A. (2003). Evaluation of backbone proton positions and dynamics in a small protein by liquid crystal NMR spectroscopy. J. Am. Chem. Soc. 125, 9179–9191. 10.1021/ja0350684 15369375

[B34] Wang X. Liu J. Zhang J. Z. H. He X. (2013). Electrostatically embedded generalized molecular fractionation with conjugate caps method for full quantum mechanical calculation of protein energy. J. Phys. Chem. A. 117, 7149–7161. 10.1021/jp400779t 23452268

[B35] Wei D. Q. Guo H. Salahub D. R. (2001). Conformational dynamics of an alanine dipeptide analog: an *ab initio* molecular dynamics study. Phys. Rev. E. 64:011907 10.1103/PhysRevE.64.011907 11461288

[B36] Weiner S. J. Kollman P. A. Case D. A. Chandra Singh U. Ghio C. Alagona G. (1984). A new force field for molecular mechanical simulation of nucleic acids and proteins. J. Am. Chem. Soc. 106, 765–784. 10.1021/ja00315a051

[B37] Weiner S. J. Kollman P. A. Nguyen D. T. Case D. A. (1986). An all atom force field for simulations of proteins and nucleic acids. J Comput. Chem. 7, 230–252. 10.1002/jcc.540070216 29160584

[B38] Wlodawer A. Walter J. Huber R. Sjolin L. (1984). Structure of bovine pancreatic trypsin inhibitor. Results of joint neutron and X-ray refinement of crystal form II. J. Mol. Biol. 180, 301–329. 10.1016/S0022-2836(84)80006-6 6210373

[B39] Wu J. C. Chattree G. Ren P. (2012). Automation of AMOEBA polarizable force field parameterization for small molecules. Theor. Chem. Acc. 131:1138 10.1007/s00214-012-1138-6 22505837 PMC3322661

[B40] Xu X. Nakatsuji H. Ehara M. Lü X. Wang N. Q. Zhang Q. E. (1998). Cluster modeling of metal oxides: the influence of the surrounding point charges on the embedded cluster. Chem. Phys. Lett. 292, 282–288. 10.1016/S0009-2614(98)00687-3

[B41] Zhang D. W. Zhang J. Z. H. (2003). Molecular fractionation with conjugate caps for full quantum mechanical calculation of protein-molecule interaction energy. J. Chem. Phys. 119, 3599–3605. 10.1063/1.1591727

